# Rewarding and Reinforcing Effects of 25H-NBOMe in Rodents

**DOI:** 10.3390/brainsci12111490

**Published:** 2022-11-02

**Authors:** Cheolmin Jo, Hyejin Joo, Dong-Hyun Youn, Jin Mook Kim, Young-Ki Hong, Na Young Lim, Kwan Soo Kim, Su-Jeong Park, Sun Ok Choi

**Affiliations:** Pharmacological Research Division, Toxicological Evaluation and Research Department, National Institute of Food and Drug Safety Evaluation, Ministry of Food and Drug Safety, Chungju 28159, Korea

**Keywords:** 25H-NBOMe, self-administration, conditioned place preference, locomotion, microdialysis

## Abstract

The drug 25H-NBOMe is a new psychoactive substance (NPS). The use of these substances is likely to pose a threat to public health because they elicit effects similar to those of known psychoactive substances with similar chemical structures. However, data regarding the abuse potential of 25H-NBOMe are lacking. Here, we evaluated the abuse liability of 25H-NBOMe in rodents. The rewarding and reinforcing effects were evaluated through conditioned place preference (CPP) and self-administration (SA) tests after administration of 25H-NBOMe. To investigate the effects of 25H-NBOMe on the central nervous system, we determined the changes in dopamine levels by in vivo microdialysis. In the locomotor activity test, 25H-NBOme significantly increased locomotor activity in mice. In the place conditioning test, the 25H-NBOMe (0.1 and 0.5 mg/kg) groups showed a significantly increase in CPP in mice. In the SA test, the 25H-NBOMe (0.01 mg/kg) administered group showed a significant increased number of infusions and active lever presses. In microdialysis, the 25H-NBOMe (10 mg/kg) administered group was significantly increased in rats.

## 1. Introduction

The United Nations Office of Drug Crime defines new psychoactive substances (NPS) as “substances of abuse, either in a pure form or a preparation, that are not controlled by the 1961 Single Convention on Narcotic Drugs or the 1971 Convention on Psychotropic Substances, but which may pose a public health threat” [1].

Recreational drugs are used to induce a change in the perception of pleasure without a medical reason, and they may be legal or illegal [2]. However, these drugs are dangerous under any circumstance and cause devastating effects, including acute toxic reactions and death, as well as social, cultural, legal, health, and political issues [3,4].

The 25X-NBOMe series (25B-NBOMe, 25C-NBOMe, 25D-NBOMe, 25E-NBOMe, 25G-NBOMe, 25H-NBOMe, 25I-NBOMe, 25N-NBOMe, and 25iP-NBOMe) are N-2-methoxybenzyl analogs of the respective 2C-X substituted phenethylamine and are reported to cause toxicity and side effects [5]. Of the 22,289 respondents of the 2013 Global Drug Survey, 582 (2.6%) had previously used NBOMe [6]. Many side effects of 25I-NBOMe, 25B-NBOMe, and 25C-NBOMe have been reported including, toxicity, tachycardia, hypertension, agitation/aggression, seizures, and high fever. However, no cases directly related to the use of 25H-NBOMe have been reported [7]. Previous studies have reported the abuse potential of 25N-NBOMe, a structural analog of 25H-NBOMe, initiated by the dopaminergic system [8]. A recent study on zebrafish embryos and larvae demonstrated the toxic effects of exposure to 25H-NBOMe over a 96-h period. The drugs 25H-NBOH and 25H-NBOMe induced high embryonic mortality at 80 and 100 µg/mL, respectively [9]. In addition, important clinical case reports on the hallucinogenic effects of NBOMes have reported severe hallucinations induced at even very low doses, and thus, they have regarded NBOMes as potentially toxic [10]. It is known that the hallucinogenic effect of NBOMes is due to the action of the serotonin 5-HT_2A_ receptor and dopamine (DA) [11].

We hypothesized that 25H-NBOMe, which belongs to the NBOMe family with reported side effects and abuse potential, would demonstrate dependence and possess abuse potential. Therefore, in this study, the rewarding and reinforcing effects and the ability of 25H-NBOMe to increase dopamine were evaluated using conditional preference (CPP), self-administration (SA) experiments, and microdialysis in rodents.

## 2. Materials and Methods

### 2.1. Animals

C57BL/6 mice (male, seven weeks, 20–23 g) and Sprague Dawley (SD) rats (male, seven weeks, 80–220 g) were purchased from Orientbio Inc. (Seongnam, Korea). All animals were maintained under a 12-h light/dark cycle (lights on at 7:00 A.M., lights off at 19:00 P.M.) and at a controlled temperature (22 ± 1 °C) and humidity (55 ± 5%) and were given ad libitum access to laboratory animal food and water. All animals were acclimated for seven days before the drug administration. The experimental groups were randomly allocated. All experimental animals were sacrificed using carbon dioxide after each experiment. All procedures pertaining to this study were approved by the National Institute of Food and Drug Safety Evaluation/Ministry of Food and Drug Safety Animal Ethics Board (approval number: MFDS-20-021/MFDS-22-020). All animal experiments complied with the National Research Council’s Guide for the Care and Use of Laboratory Animals.

### 2.2. Chemical Reagents

**Figure 1:**
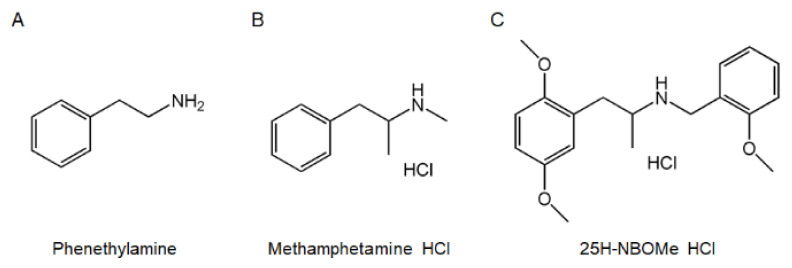
Chemical structures of phenethylamine (**A**), methamphetamine HCl (**B**), and 25H-NBOMe HCl (**C**).

**Figure 2:**
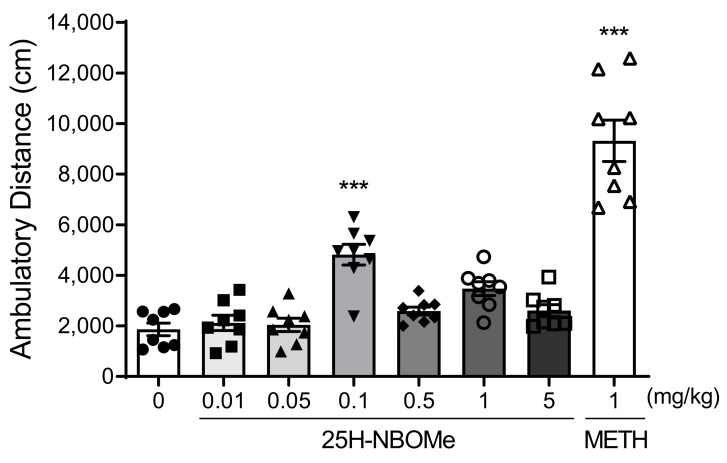
Effect of 25H-NBOMe administration on the locomotor activity in mice. Mice administered with vehicle (saline), METH (1 mg/kg), and 25H-NBOMe (0.01, 0.05, 0.1, 0.5, 1, and 5 mg/kg) were administered, and the distance traveled (cm) was measured during a 1-h test period (0: filled circles, 25H-NBOMe 0.01 mg/kg: filled squares, 25H-NBOMe 0.05 mg/kg: filled triangles, 25H-NBOMe 0.1 mg/kg: filled down triangles, 25H-NBOMe 0.5 mg/kg: filled diamonds, 25H-NBOMe 1 mg/kg: empty circles, 25H-NBOMe 5 mg/kg: empty squares, METH 1 mg/kg: empty triangles). Data are expressed as mean ± SEM (*n* = 8 each group). *** *p* < 0.001 vs. vehicle, determined by a one-way ANOVA followed by Bonferroni’s post-hoc test.

The chemical 25H-NBOMe HCl (Figure 1C), 2,5-Dimethoxy-N-[(2-methoxyphenyl)methyl]benzeneethanamine-hydrochloride (C_18_H_23_NO_3_·HCl), synthesized and purchased from Kyung Hee University (Seoul, Korea), was dissolved in a vehicle (saline). Methamphetamine (METH, Figure 1B), a strong central nervous system stimulant, was selected as the positive control since it exhibits a high potential for dependence and addiction. METH was obtained from Kyung Hee University and was dissolved in saline. Heparin was purchased from JW Pharmaceutical Corporation (Seoul, South Korea).

Entobar^®^ (Sodium pentobarbital, 100 mg/2 mL, Hanlim Pharm. Co., Ltd., Seoul, Korea) was used as an anesthetic for surgery. Ortho-Jet Liquid (B1304) and Ortho-Jet BCA powder (B1330), which act as cements for fixing the surgical site, were purchased from Lang Dental Manufacturing Co., Inc. (Wheeling, IL, USA). Artificial Cerebral Spinal Fluid (aCSF) products ACSF—ARTIFICIAL CEREBROSPINAL FLUID (1000 ML)—20X CONCENTRATE Solution A and B were purchased from Ecocyte Bioscience, Austin, TX, USA. Water for chromatography (LC-MS Grade, 1.15333.1000) and acetonitrile (1.00030.4000) used as high-pressure liquid chromatography (HPLC) solvents were purchased from Merck (Rahway, NJ, USA). Ammonium formate (516961), formic acid (#56302-1L-F), dopamine hydrochloride solution (D-081), and dopamine-D_4_ hydrochloride solution (D-072) were purchased from Sigma-Aldrich (St. Louis, MO, USA). Dopamine-D_4_ hydrochloride (DA-D_4_) was used as the internal standard (ISTD) of DA.

### 2.3. Locomotor Activity

The locomotor activity test chamber (ENV-520; Med Associates, Inc., St. Albans, VT, USA) consisted of a square plastic box (total dimensions: 43 × 43 × 31 cm^3^) with an infrared beam sensor on the floor to measure movements. For habituation, the mice (*n* = 8, each group) were allowed to move freely for 1 h in a locomotion test apparatus for 3 days without interference. On day 4 after adaptation, mice were injected intraperitoneally (i.p.) with 25H-NBOMe and 5 min later, distance traveled (cm) was recorded for 60 min. The distance traveled was automatically measured using the infrared beam sensor installed on the floor, and data were recorded using the Activity Monitor SOF-812 (Med Associates).

### 2.4. The Place Conditioning Paradigm

The place conditioning apparatus (MED-CPP-3013AT, Med Associates, Inc., St. Albans, VT, USA) consisted of three compartments, including black, white, and gray (central) compartments, separated by guillotine doors. At the start of the test, the animals were placed in the central compartment. The black room had a bar grid and the white room had a mesh grid. The size of the black and white room is 16 × 13 × 12 cm^3^. The size of the gray room (central compartment), placed between the black and white rooms, is 16 × 13 × 12 cm^3^. The illuminance of all rooms is 12 lx. The time spent in each room was recorded using an infrared sensor. The place preference test was performed as follows: On days 1 and 2, animals were allowed to move freely in all compartments for 30 min each day for habituation. After measuring pre-conditioning for 15 min on day 3, mice with more than 10% difference in the time spent in one specific room were excluded, and the remaining mice were randomly assigned. On days 4–13, vehicle (saline) or test drugs (METH 1 mg/kg or 25H-NBOMe (0.05, 0.1, and 0.5 mg/kg), i.p.) were administered on alternate days, and the mice (*n* = 8 each group) were placed in a specific room for 40 min after the administration of each test drug. Each group of experimental animals was post-conditioned for 15 min, and the time difference between post-conditioning and pre-conditioning was calculated.

### 2.5. Self-Administration

The SA test was measured in a skinner box (43 × 43 × 31 cm^3^) for conducting operant conditioning research with mice. Within this skinner box, the mouse responds by obtaining food or drugs as reinforcement using the levers (active and inactive levers). The skinner box is connected to electronic equipment (MED-307A-CT-B1; Med Associates, Inc, St. Albans, VT, USA) that records the mouse’s lever pressing, thus measuring the precise quantification of mouse behavior. A 1 mL syringe containing the drug was placed outside the skinner box and injected via an infusion pump. Food training was performed for three days; where on the first day, it was performed overnight in a skinner box and then continued for an additional two days. Mice were trained to press one of the levers for the dispensation of one food pellet (45 mg, Dustless Precision Pellets Rodent, Bio-Serv., Frenchtown, NJ, USA) placed on the food dispenser of the skinner box and delivered when the mice pressed one of the two levers (active and inactive lever). During the three days of food training, food other than that used for food training was limited to 3 g per mouse for each day. Only the mouse whose mouse response score (1 response score per lever press) was 90 or higher for three consecutive days was selected (a maximum of 100 food pellets per mouse was allowed). Only mice that met the test standard requirements received the cannulation (infusion pump) operation. Mice were anesthetized with pentobarbital (50 mg/kg, i.p.), and a catheter (26 gauge, PlasticsOne, USA) was inserted into the jugular vein and recovered for seven days. After cannulation, active lever presses, inactive lever presses, and the number of infusions of 25H-NBOMe (0.01 mg/kg), METH (0.1 mg/kg), or vehicle were measured for 2 h (fixed ratio 1 and time-out 20 s schedule) in mice (*n* = 8 each group).

### 2.6. Microdialysis

The stereotaxic apparatus used for rat brain surgery was a Model 900LS Small Animal Stereotaxic Instrument, Lazy Susan (KOPF, TUJUNGA, CA, USA). The aCSF was filtered using a 1000 mL Corning Vacuum Filter/Storage Bottle System, 0.22 μm Pore 5 (Corning, NY, USA). Syringes containing aCSF were Hamilton 1.0 mL 1001RN SYR (22/2′′/2) syringes (Reno, NV, USA). EICOM (Kyoto, Japan) products, such as a syringe pump (ESP-64), guide cannula (AG-8, length 8 mm), dummy cannula (AD-8, length 8 mm), cap nut (AC-5), anchor screw (AN-3), joint Teflon tube (JT-10), and the microdialysis probe (FX-I-8-02, shaft length 8 mm, membrane length 2 mm) were used for microdialysis. Microdialysis samples were collected in a Hard-Shell^®^ High-Profile 96-Well Semi-skirted PCR Plate (BIO-RAD, HSS9601, Contra Costa County, CA, USA) and sealed using applied biosystems MicroAmp Optical Adhesive film (ThermoFisher, Waltham, MA, USA).

The HPLC instrument used was 1260 liquid chromatography, and the tandem mass spectrometer (MS/MS) was the 6460 triple quadrupole system, both manufactured by Agilent (Santa Clara, CA, USA). The analytical column and guard column were Atlantis™ T3 3 μm (2.1 × 100 mm^2^) and ACQUITY UPLC^®^ BEH HILIC 1.7 μm VanGuard™ Pre-column (2.1 × 5 mm^2^), respectively, and were purchased from Waters Corporation (Milford, MA, USA).

#### 2.6.1. Microdialysis Surgery

Six-week-old male SD rats were acclimatized by breeding for one week. One week later, 7-week-old rats weighing 250–280 g were anesthetized by i.p. administration of sodium pentobarbital solution (50 mg/mL/kg). The hair of anesthetized rats was shaved and disinfected. Once the SD rat was fixed on a stereotaxic instrument, the scalp was incised. A hole was drilled in the SD rat skull, and the guide cannula was inserted in the nucleus accumbens coordinates [Anterior-Posterior (AP, +1.7 mm), Medial-Lateral (ML, −1.1 mm from bregma), and Dorsal-Ventral (DV, −6 mm from skull)] regions. The inserted guide cannula was fixed with dental cement, and the surgical site was sutured. A dummy cannula and a cap nut were used to protect the guide cannula from clogging until microdialysis. After surgery, the rats were subjected to microdialysis after a recovery period of 1 week or more.

#### 2.6.2. Microdialysis Sample Collection

The aCSF used for microdialysis was used by mixing solutions A, B, and distilled water in a ratio of 1:1:18 and filtering using a 0.22 μm filter. The final aCSF concentrations were as follows: pH 7.4; NaCl 125 mM, KCl 3 mM, CaCl_2_ 2.5 mM, MgSO_4_ 1.3 mM, NaH_2_PO_4_ 1.25 mM, NaHCO_3_ 26 mM, and glucose 13 mM. A 1 mL Hamilton syringe containing aCSF was installed in the syringe pump, and aCSF was allowed to flow at a rate of 2 μL per minute. A syringe was connected to one side of the Teflon tube, and a microdialysis probe inlet was connected to the other side of the tube. The dummy cannula in the guide cannula inserted from the head of a 7-week-old rat was removed, and a probe was inserted in the guide cannula and fixed with a cap nut. The sample collector was connected to the other Teflon tube at the probe outlet side. A 96-well plate was mounted on the sample collector, and the aCSF dialyzed through the probe was collected. The collector was kept at 4 °C. The aCSF collected during the first hour of stabilization was discarded. After 1 h, baseline samples were collected three times in a 96-well plate using a collector at 20-min intervals. After all baseline samples were collected, the substances to be used for the experiment (25H-NBOMe HCl 0, 5, 10 mg/kg, METH 5 mg/kg) were i.p. administered to different rats (*n* = 3–4 each group). After administration, samples were collected in 96-well plates for 2 h at 20-min intervals. After sample collection, the 96-well plate was sealed with film. Samples were immediately analyzed by LC-MS/MS or stored at −80 °C until analysis to prevent DA deterioration.

#### 2.6.3. Dopamine Analysis of Microdialysis Samples by LC-MS/MS

To 30 μL of the microdialysis sample, 10 μL of DA-D_4_ HCl in distilled water (ISTD, 20 ppb) was added, mixed, and stored in an amber LC vial. The sample vials were maintained at a temperature of 4 °C in the autosampler to prevent the deterioration of DA. Each sample mixture (10 μL) was injected into LC-MS/MS, and multiple reaction monitoring (MRM) conditions were set according to a previous study [12]. The LC solvent A was 2 mM ammonium formate + 0.1% formic acid in MS grade water, whereas the LC solvent B was 0.1% formic acid in acetonitrile. The total solvent flow was fixed at 0.3 mL/min, and the column oven temperature was fixed at 35 °C. The initial solvent composition was maintained at 0% of solvent B for 1 min, then changed linearly to 50% (1–4.5 min) and 80% (4.5–5 min). Then 80% of solvent B was held for 1 min (5–6 min). This was followed by returning to the initial conditions within 0.1 min (6–6.1 min) and holding for 2.5 min (6.1–10 min) for re-equilibration. In order to prevent device contamination by salts and other impurities in the sample, the solvent that passed through the column for 1 min after sample injection was not allowed to enter the MS/MS part.

#### 2.6.4. Data Processing

The DA concentration for each sample was calculated using a quantitative analysis program (Masshunter Quantitative Analysis ver. B.06.00, Agilent, Santa Clara, CA, USA) for the chromatograms collected after mass spectrometry. The DA concentration was converted to DA% of the basal level in order to reduce the difference between each rat. The conversion was completed through the following equation:

$$\mathrm{DA}\;\left(\%\;\mathrm{of}\;\mathrm{Basal}\;\mathrm{level}\right)=\frac{\mathrm{DA}\;\mathrm{conc}.\;\mathrm{in}\;\mathrm{each}\;\mathrm{sample}\;\mathrm{collected}\;\mathrm{for}\;20\;\min\;}{\mathrm{Average}\;\mathrm{of}\;\mathrm{DA}\;\mathrm{conc}.\mathrm{of}\;3\;\mathrm{baseline}\;\mathrm{samples}\;}\times 100$$

### 2.7. Data Analysis and Statistics

All data were randomized and performed in a blinded manner. All experimental data are represented as mean ± standard error of the mean (SEM), and statistical analysis was performed using the Graph-Pad Prism (GraphPad Software 8, Inc. San Diego, CA, USA). The locomotor activity data were analyzed using one-way ANOVA and a Bonferroni post-hoc test. The CPP test and SA test data were analyzed using an ordinary two-way ANOVA and Bonferroni post-hoc test, and the microdialysis data were analyzed using an ordinary two-way ANOVA and Dunnett post-hoc test. *, **, and *** in the figures denote *p* < 0.05, 0.01, and 0.001, respectively.

## 3. Results

### 3.1. Locomotor Activity Stimulation by 25H-NBOMe

The distance traveled (cm) during the 1 h test period 5 min after drug administration was greater in the METH and 25H-NBOMe treatment groups when compared to the vehicle group. The 25H-NBOMe (0.01, and 0.05 mg/kg) groups did not differ significantly in their ambulatory activity from the vehicle group. In the 25H-NBOMe 0.1 mg/kg dose group, locomotor activity was significantly increased, and at higher doses (0.5–5 mg/kg), locomotor activity decreased (Figure 2). The activity increased to a maximum at a specific dose and then decreased, possibly due to the characteristic bell-shaped dose-response curve induced by hallucinogens.

### 3.2. 25H-NBOMe Induced Conditioned Place Preference (CPP)

**Figure 3:**
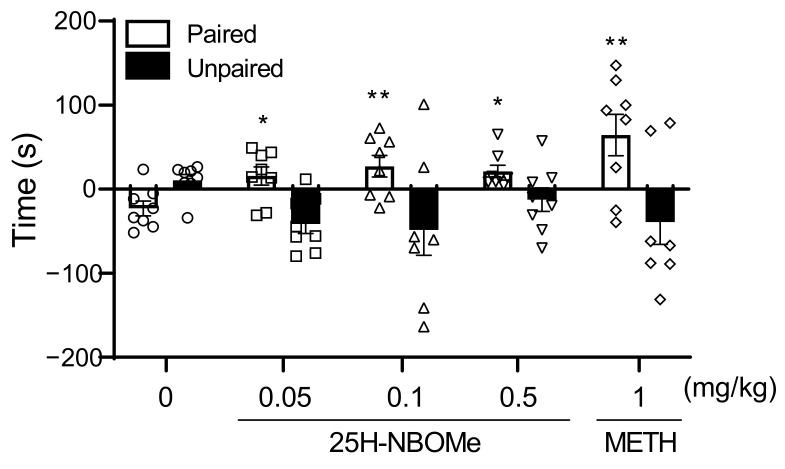
Effect of 25H-NBOMe administration on the place conditioning paradigm in mice. Time difference spent in the drug-paired (white bar) and drug-unpaired (black bar) compartment between the drug administration group and the vehicle (saline) group during pre-conditioning and post-conditioning (0: circles, 25H-NBOMe 0.05 mg/kg: squares, 25H-NBOMe 0.1 mg/kg: triangles, 25H-NBOMe 0.5 mg/kg: down triangles, METH 1 mg/kg: diamonds). Data are expressed as mean ± SEM (*n* = 8 each group). * *p* < 0.05; ** *p* < 0.01 vs. vehicle, determined by a two-way ANOVA followed by Bonferroni’s post-hoc test.

The METH (1 mg/kg) group showed the highest place preference (time) compared to other groups. The 25H-NBOMe 0.05, 0.1, and 0.5 mg/kg groups expressed a significant place preference (time) compared to the vehicle group (Figure 3).

### 3.3. Mice Self-Administered 25H-NBOMe

**Figure 4:**
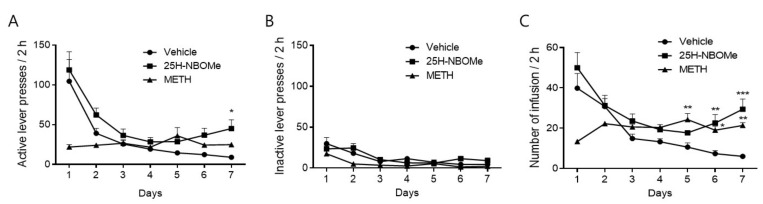
Intravenous self-administration (SA) of 25H-NBOMe and methamphetamine (METH). Data are expressed as the mean number of active lever presses (**A**), inactive lever presses (**B**), and infusion (**C**), following SA of 25H-NBOMe (0.01 mg/kg) and METH (0.1 mg/kg). Data are expressed as mean ± SEM (*n* = 8 each group). * *p* < 0.05, ** *p* < 0.01, *** *p* < 0.001 vs. vehicle, determined by a two-way ANOVA followed by Bonferroni’s post-hoc test.

The SA test was conducted for 2 h/session per day with a fixed ratio of 1 and a time-out schedule of 20 s. Mice having access to METH (0.1 mg/kg/infusion, i.v.) and 25H-NBOMe (0.01 mg/kg/infusion, i.v.) exhibited increased activity of lever presses on days 5, 6, and 7 compared to activity exhibited by the vehicle group. Moreover, mice having access to 25H-NBOMe exhibited significantly increased active lever presses on day 7 (Figure 4A). There was no significant difference in the number of inactive lever presses between all groups (Figure 4B). In both the 25H-NBOMe and the METH groups, the number of infusions increased significantly compared to that in the vehicle group (Figure 4C).

### 3.4. Changes in DA Levels in the In Vivo Microdialysis Test

**Figure 5:**
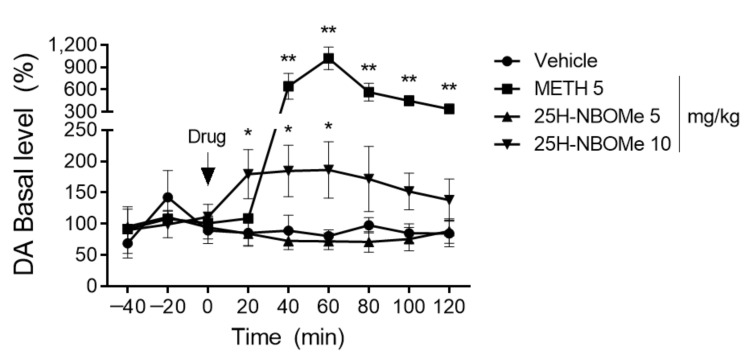
The dopamine (DA) levels in the striatum after i.p. administration of vehicle (saline), METH (5 mg/kg), and 25H-NBOMe (5 and 10 mg/kg). The black arrow indicates when the drug is administered. Data are expressed as mean ± SEM (*n* = 3–4 each group). * *p* < 0.05, ** *p* < 0.01 vs. vehicle, determined by a two-way ANOVA followed by Dunnett’s post-hoc test.

To investigate whether 25H-NBOMe regulates extracellular DA levels in the striatum, we intraperitoneally injected 5 and 10 mg/kg of 25H-NBOMe into rats and sampled DA every 20 min for 120 min. During the 40 min before 25H-NBOMe administration, there was no significant change in DA levels in any of the groups administered with 25H-NBOMe compared with that in the vehicle group. However, the basal DA level increased significantly 40 min after the administration of 5 mg/kg METH, reached a peak at 60 min, and then gradually decreased. After administration of 5 mg/kg 25H-NBOMe, there was no change in DA levels; however, the DA levels significantly increased 60 min after administration of 10 mg/kg 25H-NBOMe (Figure 5).

## 4. Discussion

Although the abuse of drugs is strongly controlled and prohibited, the emergence of NPS is a new threat to human health worldwide. NPS is synthesized by making minor modifications to the structures of well-known drugs while avoiding current regulations; their psychoactive, stimulant, and hallucinogenic effects are the same as or greater than those of the parent drugs. The present study provides evidence for the danger of NPS as a narcotic by identifying the scientific basis for the abuse potential of 25H-NBOMe, an NPS.

NBOMes are phenylethylamine hallucinogens with a high affinity for the 5-HT2A receptor [13] and are structurally similar to the amphetamine class (EMBL-EBI. CHEBI:191069-Benzeneethanamine, 2,5-dimethoxy-n-[(2-methoxyphenyl)methylene] [14]). These hallucinogens mainly regulate serotonin levels more effectively than DA levels [15]. However, recent studies report that hallucinogens have a major effect on the DA system as well as the serotonin system [16]. 

Several DA pathways exist in the brain, one of which plays an important role in reward-motivated behavior [17,18]. Reinforcing effects appear when DA levels in the brain increase, and the abuse liability of most addictive drugs is dependent on increasing DA levels [19]. Therefore, we measured DA levels by microdialysis to confirm the association between reward and reinforcing effects of 25H-NBOMe and DA, using the structurally similar METH as a positive control.

METH and 25H-NBOMe belong to the class of amphetamines that have a phenethylamine structure (EMBL-EBI. CHEBI:6809-Methamphetamine [20]). METH increases neurotransmitter levels such as DA, norepinephrine, and serotonin [21]. Specifically, METH use releases DA into the synaptic cleft, thereby increasing DA levels, but inhibits the movement of DA into storage vesicles, increasing synaptic DA concentrations [22,23]. DA increases locomotor activity and is strongly associated with enthusiasm, motivation, control, and rewarding and reinforcing behavior [24,25]. Euphoria also correlates with the release of DA [26]. Thus, METH activates the reward system of the brain, causing highly reinforcing effects that lead to its abuse and dependence. Previous studies have reported that METH, like many amphetamine class drugs, increases DA levels, resulting in its dependence and potentiation effects [27,28]. Therefore, we hypothesized that 25H-NBOMe, being structurally similar to METH, would also show dependence and abuse potential due to the lack of preclinical and clinical data on its toxicity and dependence.

To test our hypothesis, we evaluated the abuse liability of 25H-NBOMe in mice by assessing its rewarding and reinforcing effects through CPP tests and SA tests. The CPP and SA experimental paradigms are the most common methods to prove rewarding and reinforcing effects, respectively, in experimental animal models [29,30]. Moreover, as the reinforcing effects and locomotor activity may share similar neuronal processes [31], we analyzed locomotor activity (ambulatory behavior).

We found that administration of 0.05, 0.1, and 0.5 mg/kg i.p. 25H-NBOMe caused place preference in the CPP test, and a similar trend was observed for 1 mg/kg METH administration. These results suggest that 25H-NBOMe may have a rewarding effect. In addition, the administration of 0.1 mg/kg of 25H-NBOMe had a significant effect on the CPP score and distance traveled, indicating psychological and physical activity. In contrast, 0.5 mg/kg of 25H-NBOMe administration showed a significant effect on the CPP score but had no significant effect on locomotor activity. These results suggest that preference for a place is not due to an increase or change in motor activity. In the SA test, the number of infusions was significantly increased in the 25H-NBOMe (0.01 mg/kg/infusion) group compared with the vehicle group on days 6 and 7. The reinforcing effects observed were higher than those of the group administered with 0.1 mg/kg METH on day 7. To evaluate the association of these behavioral changes with DA, we measured DA levels in the rat brain striatum by in vivo microdialysis. Although the DA levels in the 10 mg/kg 25H-NBOMe group were not as high as those in the 5 mg/kg METH group, the 10 mg/kg 25H-NBOMe group showed a significant increase in DA basal levels immediately after administration and remained elevated up to 40 min post-administration. This finding is noteworthy as it suggests that hallucinogens may affect the dopaminergic system.

## 5. Conclusions

Administration of 25H-NBOMe induced rewarding and reinforcing effects in mice and increased extracellular dopamine in the striatum in rats. However, the results obtained cannot be described as a direct effect of increased dopamine levels because re-warding and reinforcing effects and dopamine level measurements differ based on the animals and dose. In addition, only male rodents were used in this study, and the number of animals used in the microdialysis test was small. This is the first animal study to evaluate the dependence and abuse potential of 25H-NBOMe. Our findings provide scientific evidence for the regulation of this NPS. However, this study was limited to in vivo studies, and the effects of 25H-NBOMe cannot be generalized to clinical practice. Thus, this study may have potential impacts on the policy for the regulation of narcotics; further related mechanistic and toxicity studies are needed in the future.

## Data Availability

Not applicable.

## Abbreviations

ANOVA	analysis of variance
CPP	conditioned place preference
DA	dopamine
METH	methamphetamine hydrochloride
NPS	new psychoactive substance
SA	self-administration
SD	Sprague–Dawley
